# Implementation process of a large multicenter study in trauma

**DOI:** 10.1186/cc14536

**Published:** 2015-03-16

**Authors:** AF Turgeon, TBI-Prognosis Team in collaboration with the CCCTG

**Affiliations:** 1 CHU de Québec, QC, Canada

## Introduction

Our purpose was to evaluate the time from shipping of the study start-up package to study screening, as well as conditions that may impact this process, in the context of a large-scale multifaceted and multicenter clinical study in trauma.

## Methods

We designed a survey questionnaire based on four domains: REB characteristics and process, centers' characteristics, experience of the study and clinical teams, and center-specific implementation approaches. The questionnaire was self-administered to all lead research coordinators of the 17 level 1 participating trauma centers in the ongoing Pan-Canadian TBI-Prognosis Study. Descriptive statistics were used.

## Results

Overall, 33.4 weeks (95% CI = 24.8 to 42.1) were required on average from the start-up package mailing to centers and the start of the screening process. Data sharing and financial agreement were mainly responsible for this delay with an average of 28.6 weeks (95% CI = 20.4 to 36.7) needed to complete the agreement with the coordinating center. REB approval was obtained on average 17.5 weeks (95% CI = 13.8 to 21.2) from the shipping of the study package to the participating centers. Eighty percent of the REBs had members with prior experience in multicenter clinical research, and more than half with specific clinical expertise in critical care medicine or neurology/ neurosurgery. A standardized electronic REB submission process was used in most REBs (60%). All centers had experience in implementing multicenter clinical studies. PIs had experience conducting from zero to 40 prior clinical studies and from 3 to 24 years of research experience with protected research time ranging from 5 to 75%. RCs had managed from zero to more than 50 clinical studies. Most centers (87%) organized specific presentation of the study to the critical care medical staff (93%), while some (60%) presented the study to other medical teams. Agreements from other departments such as radiology (87% of centers), electrophysiology (80%) and clinical imaging (73%) were requested in most centers.

## Conclusion

The implementation of a large Canadian multicenter and multifaceted clinical study in the trauma population involved a significant amount of time and energy from both the coordinating and the participating sites. The variable experience of participating sites and teams, as well as the involvement of different medical disciplines, may have had an impact on study implementation time. Delays for REB approval but also for data sharing and financial agreement must be taken into consideration in the timeline for implementing large multicenter clinical studies in trauma.

